# Neuroprotective Effects of Estradiol plus Lithium Chloride via Anti-Apoptosis and Neurogenesis Pathway in *In Vitro* and *In Vivo* Parkinson's Disease Models

**DOI:** 10.1155/2021/3064892

**Published:** 2021-10-22

**Authors:** Yung-Shih Lee, Chien-Wei Feng, Mei-Yu Peng, Te-Fu Chan, Yu-Chieh Chen

**Affiliations:** ^1^Department of Obstetrics and Gynecology, Kaohsiung Medical University Hospital, Kaohsiung 807377, Taiwan; ^2^Center for Cancer Research, Kaohsiung Medical University, Kaohsiung 807377, Taiwan

## Abstract

Few pharmaceutical agents for slowing Parkinson's disease (PD) progression existed, especially for perimenopause females. The current general medications are mostly hormone replacement therapy and may have some side effects. Therefore, there is an urgent need for a novel treatment for PD. This study examined the possibility of estradiol plus lithium chloride (LiCl), one of the metal halides used as an alternative to salt. We showed that the combination of LiCl and estradiol could enhance neurogenesis proteins GAP-43 and N-myc in the human neuronal-like cells. We also further confirmed the neurogenesis activity in zebrafish. LiCl and LiCl plus estradiol could enhance 6-OHDA-induced upregulation of TGase-2b and Rho A mRNA expression. Besides, LiCl plus estradiol showed a synergic effect in anti-apoptotic activity. LiCl plus estradiol protected SH-SY5Y cells and zebrafish against 6-OHDA-induced damage on neurons than LiCl or estradiol alone groups via p-P38, p-Akt, Bcl-2, and caspase-3 cascade. The potential for developing this combination as a candidate treatment for PD is discussed.

## 1. Introduction

Parkinson's disease (PD) is one of the most complex neurodegenerative diseases in the world. By gender, women were observed with a less frequency than in men, about 1 : 1.5. However, Picillo et al. demonstrated that perimenopause might increase the possibility of developing a neurodegenerative disease in female patients [1]. Hence, hormone therapy has become a choice in perimenopause patients. The first study that revealed the neuroprotective role of estrogen came from Hall et al. This study examined the therapeutic efficacy of estrogen in different gender of traumatic brain injury models, and the result showed that female gerbils suffered less than male gerbils [2]. Some further studies used exogenous estrogen to inhibit the volume of thrombosis in the ischemia rat model [3, 4]. Gibson et al. systemically reviewed 161 original articles, including 1304 individuals, and revealed that estrogen had a significant therapeutic effect in neurodegenerative animal models [5]. Then, hormone replacement therapy (HRT) began to be used in patients.

Nowadays, the application of HRT is used chiefly on perimenopause women and combined with progesterone. There were some advantages of HRT. First, it could relieve the symptoms of menopausal syndrome, which helped improve their life quality. In addition, HRT could decrease the incidence of cardiovascular disease in perimenopause women [6]. Some studies also proved that estrogen could improve the symptoms by reducing low-density lipoprotein (LDL) [7, 8]. HRT could also prevent osteoporosis by activating the osteoblast's maturation and inhibiting the osteoclast [9]. However, previous studies revealed that some severe adverse effects showed up after the treatment of HRT. Rossouw et al. demonstrated that HRT could slightly increase the incidence of breast cancer [10]. Besides, Anderson et al. found that HRT could increase the possibility of septic shock in perimenopausal women [11]. Then, Hampton et al. also depicted that HRT would enhance the death rate of lung cancer patients [12]. Due to the adverse effects mentioned above, some studies attempted to combine other agents to reduce the dose of estrogen and further improve menopausal symptoms.

Wimalawansa et al. showed that the combination of estrogen and etidronate could increase patients' bone mineral density (BMD) by about 10.9% in vertebrae and 7.25% in femora in 4 years of treatment compared to the placebo group, respectively. The patients treated with etidronate alone increase by 6.79% and 1.2%, and those treated with estrogen alone increase by 6.78% and 4.01% [13]. Kinn et al. also depicted that the combination of estrogen and phenylpropanolamine could significantly improve symptoms in patients, such as subjective appraisal, micturition schedules, pad-test, and urodynamic methods than either drug alone [14]. Geusens et al. also showed that the osteogenesis effect of combining vitamin D with estrogens is more effective than estrogens alone. They also conclude that the treatment of estrogens could prevent bone loss from ovariectomy in female rats. These studies showed that the feasibility of the combination of estrogen with other agents. They only focused on the osteogenesis effect of estrogen with some compounds. Furthermore, some studies investigated the neuroprotective activity of the combination of estrogen and other agents in experimental models, such as estrogen plus agomelatine, estrogen plus dihydrotestosterone, or estradiol plus testosterone [15–19]. Although these combinations did not apply to clinical PD treatment, none of them were used at the bedside. Thus, our studies intend to find a new combination to improve its neuroprotective effect.

Lithium chloride (LiCl) is one metal halide that was used as an alternative to salt. LiCl was also used as a medication for bipolar disorders [20]. This indication also demonstrated that LiCl might have the ability to cross the blood-brain barrier (BBB). Previous studies also support this hypothesis [21, 22]. Some research then investigated the neuroprotective activity of LiCl. Fan et al. depicted that treatment of LiCl can improve the pathological changes and cognitive outcome via upregulating p-Akt and p-GSK3*β* expressions in repeated ischemia-reperfusion brain injury [23]. Liu et al. demonstrated that administration of LiCl could attenuate sensorimotor dysfunction and cognitive impairment in intracerebral hemorrhage rats through inhibition of GSK-3*β* pathway and NF-*κ*B pathway [24]. Boyko et al. also indicated that LiCl could significantly attenuate interleukin-6 and prostaglandin E2 in the rat septic shock model [25]. Doeppner et al. also found that LiCl treatment alleviated the infiltration of white blood cells and activation of microglia cells in the rat septic shock model [26]. The studies mentioned above revealed the possibility of LiCl in the co-treatment of estrogen.

Previous literature demonstrated that some clinical PD drugs, such as rasagiline, amantadine, and talipexole, have been shown to confer neuroprotective activity in the SH-SY5Y cell model with neurotoxins, such as 6-OHDA or 1-methyl-4-phenylpyridinium (MPP^+^) [27–30]. Even the recent study made by Varier et al. used amantadine as positive control in 6-hydroxydopamine- (6-OHDA-) treated SH-SY5Y model to assess the neuroprotective effect with a tropolone-related compound, Hinokitiol. Hence, we evaluated the neuroprotective activity in 6-OHDA-treated SH-SY5Y cell model. Furthermore, many studies have indicated that the administration of 6-hydroxydopamine to zebrafish specifically destroys the dopamine neurons [31–35]. The study made by Feng et al. used zebrafish as a model to examine some potential compounds or clinical drugs, such as vitamin E, minocycline, and even Sinemet®, to confirm the feasibility of the 6-OHDA-induced PD model [36]. Besides, Cronin et al. showed that zebrafish could reflect preliminary clinical findings for rasagiline and minocycline. Hence, they thought a zebrafish model is suitable for high-throughput screening of putative neuroprotective and neuro-restorative therapies to treat Parkinson's disease [35]. The studies mentioned above revealed that the 6-OHDA-induced zebrafish PD model might provide helpful information on assessing the neuroprotective effect. Thus, our study intended to examine the therapeutic effects of combined estrogen and lithium chloride therapy in these two models. We aimed to confirm the mechanism of action of combined therapy and examine the efficacy of combined therapy with these two agents, respectively. We hope our study could benefit the current treatment situation.

## 2. Materials and Methods

### 2.1. Cell Lines

The SH-SY5Y cell line was purchased from American Type Culture Collection (ATCC, No. CRL-2266). The cells were incubated with Dulbecco's Modified Eagle Medium (DMEM) (Invitrogen Corporation, Carlsbad, CA, USA) in a 5% CO_2_ 37°C incubator. Cell numbers for each assay were listed as follows: cell survival assay (trypan blue): 1.8 × 10^6^/well for 6-well microplates (passage 21–24); cell survival assay (AlamarBlue assay): 3 × 10^4^/well for 96-well microplates, 1.8 × 10^6^/well in 6-well culture plate containing coverslips (24 × 24 mm) for TUNEL staining; and 1 × 10^6^/dish in the 10 cm dish for Western blot analysis.

### 2.2. Cell Viability

We examined the cell viability with two methods as follows. The cultured cells were incubated with 100 nM *β*-estradiol (E2) and different concentrations (1 and 5 mM) of lithium chloride (LiCl) for 1 hr before 18-hour incubation of 6-hydroxydopamine (6-OHDA) (Sigma, St. Louis, MO, USA). Then, 10 *μ*l AlamarBlue (Invitrogen, Waltham, MA, USA) in each well was added and measured by ELISA reader (595 nM). Cell viability was calculated as 100 × [(optical density (OD) of 6-OHDA/LiCl plus E2-treated cells − OD of blank-treated cells)/(OD of control cells − OD of blank-treated cells)]. Another method was using trypan blue assay. The cultured cells were treated for 100 nM E2 and 1 mM of LiCl for 1 hr before 18-hour treatment of 20 *μ*M 6-OHDA. The cell numbers were counted by bright field microscopy with 0.4% trypan blue. 10 *μ*l cell suspension was mixed with 10 *μ*l trypan blue and put in a counting chamber. The final concentrations we used in our study are listed below: LiCl (1 or 5 mM), E2 (100 nM), and 6-OHDA (20 *μ*M) in the SH-SY5Y cell model; LiCl (1 mM), E2 (100 nM), and 6-OHDA (250 *μ*M) in the zebrafish model. The 6-OHDA concentrations we used in *in vitro* and zebrafish model can be referred to in our previous literature [37, 38]. The LiCl concentrations we used in the *in vitro* model can be referred to in Hou et al. [39], and the E2 concentration we used in the *in vitro* model can be referred to in Pan et al. [40]. The LiCl and E2 concentrations we used in zebrafish model came from our own test (data not shown).

### 2.3. TUNEL Stain

The SH-SY5Y cells in 6-well microplates were treated with 100 nM E2 and 1 mM of LiCl for 1 hr before 8-hour treatment of 20 *μ*M 6-OHDA. Then, we used *in situ* cell death detection kit, POD (Roache Diagnostics, Germany), to assess the cell apoptosis according to the manufacturer. In brief, we washed cells with phosphate-buffered saline (PBS) (NaCl (137 *μ*M), KCl (2.7 mM), Na2HPO4 (10 mM), and KH2PO4 (1.8 mM)) twice and fixed them with 4% paraformaldehyde. Then, the cells were blocked by 3% H_2_O_2_ of methanol and stained with a TUNEL reaction mixture (with DAPI) in a 37°C CO_2_ incubator. We then observed the signal by fluorescence microscopy. Apoptotic cells were validated by fluorescent signal with a microscope (Olympus, Tokyo, Japan, IX-51), and the ratio of apoptotic cells was measured as follows: number of apoptotic cells/total cell number from three random fluorescent images.

### 2.4. Western Blotting

The cells and zebrafish were treated in 6 cm dish as designed. In cell, the cell pellet was collected and lysed with an equal volume of lysis buffer (Tris-HCl (10 mM, pH = 8.0), EDTA (1 mM), EGTA (0.5 mM), Triton X-100 (1%), sodium deoxycholate (0.1%), SDS (0.1%), and NaCl (140 mM) contained 0.1% protease inhibitor cocktail). The obtained protein was then adjusted to the same concentration with Bradford protein assay and added sample buffer (SDS (2%), glycerol (10%), bromophenol blue (0.1%), 2-mercaptoethanol (2%), and Tris-HCl (50 mM), pH = 7.2). The prepared protein samples (approximately 100 *μ*g for each sample) were processed by the SDS-PAGE electrophoresis separation. The target proteins were then transferred to an activated PVDF membrane. The PVDF membrane was first blocked with 5% non-fat dry milk in Tris-buffered and then incubated with the primary antibody overnight at 4°C. A horseradish peroxidase-conjugated secondary antibody was detected by chemiluminescence (Millipore Corp., Billerica, MA, USA). Images were obtained using the UVP BioChemi Imaging System, and LabWorks 4.0 software (UVP) was used to quantify the relative densitometry. The relative density of protein expressions was normalized by the internal control *β*-actin.

### 2.5. Zebrafish Maintenance

We used wild-type zebrafish (AB strain) in this study. The embryos were obtained through natural spawning and screened at the typical stages by standard criteria [41]. The embryos were incubated at 28.5°C in Hank's buffer (NaCl (13.7 mM), KCl (540 *μ*M), Na_2_HPO_4_ (25 *μ*M), KH_2_PO_4_ (44 *μ*M), CaCl_2_ (300 *μ*M), MgSO_4_ (100 *μ*M), and NaHCO_3_ (420 *μ*M)).

### 2.6. The 6-OHDA-Induced Zebrafish PD Model

6-OHDA dissolved in 2% freshly prepared L-ascorbic acid (Sigma, St. Louis, MO, USA) [42]. LiCl (1 or 5 mM) and E2 (100 nM) were treated from 9 hours postfertilization (hpf) to 5 days postfertilization (dpf) in 24-well microplates. We then co-treated 6-OHDA (250 *μ*M) from 2 to 5 dpf. The qPCR and protein sample were obtained at 3 dpf and 5 dpf, respectively. The protocol of zebrafish behavior can be referred to in a previous study [36]. In brief, zebrafish larvae were put into the cuvette, and then their behavior was monitored by an automated video tracking system (Singa Technology Co.; catalog no. TM-01). The cuvette size used in this study was 1-1-4.5 cm (L-W-H) and put in front of the camera for 7.5 cm. Every zebrafish in the test was given a 2 min adaptation time; then the swimming pattern and swimming total distance of each fish was recorded for 5 min.

### 2.7. Zebrafish RNA Extraction and Quantitative Real-Time Polymerase Chain Reaction

Zebrafish embryos were treated for 87 hr with LiCl (1 or 5 mM) and E2 (100 nM) in the presence or absence of 6-OHDA. Total RNA was extracted from 20 zebrafish larvae of each treatment group using the TRIzol_Reagent (Invitrogen, Waltham, MA, USA). RNA was reverse-transcribed to single-stranded cDNA using the iScript cDNA synthesis kit (Bio-Rad, Hercules, CA, USA; catalog no. 1708891). The following reverse transcription-polymerase chain reaction (RT-PCR) using the gene expression assay primer for zebrafish was used.

Primers are as follows:

  BDNF: forward: 5″- ATAGTAACGAACAGGATGG -3″
  reverse: 5″- GCTCAGTCATGGGAGTCC -3″;
  Bcl-2: forward: 5″-TCAATAAAGCAGTGGAGGAATC-3″
  reverse: 5″- TCAAATGAGGGTCTGAACGAG -3″;
  Rho A: forward: 5″-TCCTGAGGTTTACGTTCCCA-3″
  reverse: 5″- TGGCCAGCTGTATCCCATAG-3″;
  TGase: forward: 5″-CTCGTGGAGCCAGTTATCAACAGCTAC-3″
  reverse: 5″- TCTCGAAGTTCACCACCAGCTTGTG-3″;
  Caspase-3: forward: 5″-AGGACTCTAGACGGCATCCA-3″
  reverse: 5″-CAGTGAGACTTGGTGCAGTG-3″
  GAPDH: forward: 5″-GAAGGTGAAGGTCGGAGTC -3″
  reverse: 5″- GAAGATGGTGATGGGATTTC -3″.

We then performed real-time PCR using the Fast SYBR™ Green Master Mix (Bio-Rad, Hercules, CA, USA; catalog no. 4385612) for zebrafish in the Bio-Rad real-time PCR system. The expression level of each gene was expressed as a relative fold change (log2 ratio) that was calculated using the comparative Ct method and GAPDH as the internal reference.

### 2.8. Chemicals and Antibodies

1. 6-Hydroxydopamine (6-OHDA) (Sigma, St. Louis, MO, USA; catalog: H4381)
2. *β*-Estradiol (E2) (Sigma, St. Louis, MO, USA; catalog: E8875)
3. Lithium chloride (LiCl) (Sigma, St. Louis, MO, USA; catalog: L9650)
4. AlamarBlue (Invitrogen, Waltham, MA, USA; catalog: 786–922)
5. *β*-Actin (loading control; Sigma, St. Louis, MO, USA; catalog: A5441; 1 : 3000 dilution)
6. p-ERK (Cell Signaling Technology, Danvers, MA, USA, Thr202/204; catalog: 9101; 1 : 1000 dilution)
7. ERK (Cell Signaling Technology, Danvers, MA, USA; catalog: 9102; 1 : 1000 dilution)
8. p-P38 (Cell Signaling Technology, Danvers, MA, USA; Thr180/Thr182, catalog: 9211; 1 : 1000 dilution)
9. P38 (Cell Signaling Technology, Danvers, MA, USA; catalog: 9212; 1 : 1000 dilution)
10. p-Akt (Cell Signaling Technology, Danvers, MA, USA; catalog: 9271; 1 : 1000 dilution)
11. Akt (Cell Signaling Technology, Danvers, MA, USA; catalog: 9272; 1 : 1000 dilution)
12. Growth-associated protein 43 (GAP-43) (Sigma-Aldrich, St. Louis, MO, USA; catalog: 951421273; 1 : 1000 dilution)
13. N-myc (Thermo Fisher Scientific Inc., Waltham, MA, USA; catalog: PIPA5117717; 1 : 1000 dilution)
14. Tyrosine hydroxylase (TH) (Millipore Corp., Billerica, MA, USA; catalog: Mab318; 1 : 1000 dilution)

### 2.9. Statistical Analysis

Results were presented as mean ± SEM. For western blotting data, the intensity of each band was expressed as the relative integrated density divided by the average integrated density values from all internal controls. Data were analyzed using one-way analysis of variance followed by Tukey's test. *p* value less than 0.05 was considered statistically significant.

## 3. Results

### 3.1. Effect of Lithium Chloride Plus Estradiol on 6-OHDA-Induced Apoptosis in SH-SY5Y Neuroblastoma Cell Line

**Figure 1:**
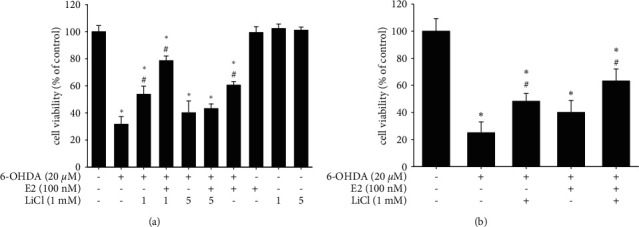
Effect of estradiol (E2) plus lithium chloride (LiCl) on 6-OHDA-induced SH-SY5Y cell death. (a) The SH-SY5Y cells were pretreated with 1 or 5 mM LiCl for 1 hr in the absence or presence of 100 nM E2 and then challenged with 20 *μ*M 6-OHDA for 16 hr. Cell viability of control, 6-OHDA, 6-OHDA plus 1 mM LiCl, 6-OHDA plus 1 mM LiCl and 100 nM E2, 6-OHDA plus 5 mM LiCl, 6-OHDA plus 5 mM LiCl and 100 nM E2, 6-OHDA plus 100 nM E2, 100 nM E2 alone, 1 mM LiCl alone and 5 mM LiCl alone. Cell viability was assessed by AlamarBlue. (b) The SH-SY5Y cells were pretreated with 1 mM LiCl for 1 hr in the absence or presence of 100 nM of E2 and then challenged with 20 *μ*M 6-OHDA for 16 hr. Cell viability of control, 6-OHDA, 6-OHDA plus 1 mM LiCl, 6-OHDA plus 1 mM LiCl and 100 nM E2, and 6-OHDA plus 100 nM E2 by trypan blue. The value corresponds to the mean ± SEM of five independent experiments that have been done in triplicate (*n* = 15). ^*∗*^*p* < 0.05 compared with the control group; ^#^*p* < 0.05 compared with the 6-OHDA group.

**Figure 2:**
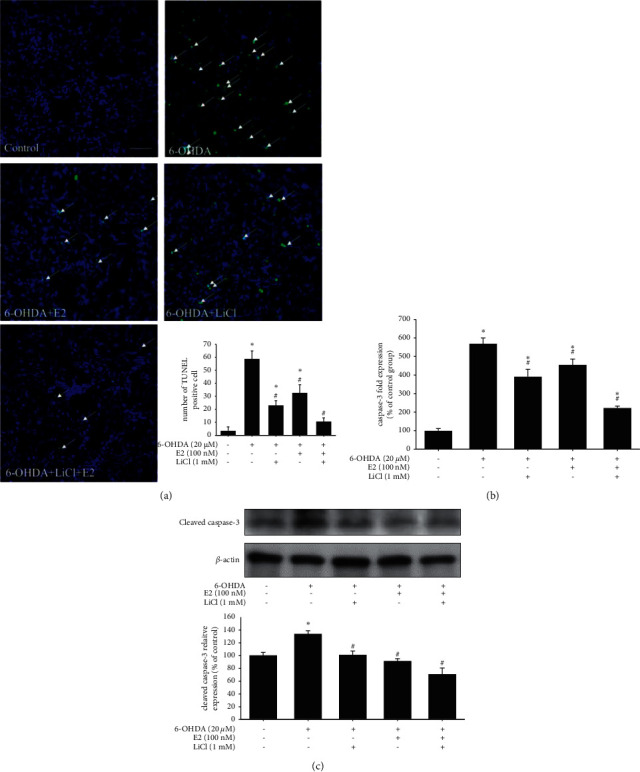
The anti-apoptosis effect of E2 plus lithium chloride on 6-OHDA-induced damage in SH-SY5Y cells. (a) The SH-SY5Y cells were pretreated with 1 mM LiCl for 1 hr in the absence or presence of 100 nM E2 and then challenged with 20 *μ*M 6-OHDA for 8 hr. Apoptotic effect of control, 6-OHDA, 6-OHDA plus 1 mM LiCl, 6-OHDA plus 1 mM LiCl, and 100 nM E2 and 6-OHDA plus 100 nM E2 groups. TUNEL staining was performed, and white arrows represent apoptotic cells (scale bar, 100 *μ*m). The quantification result of apoptotic cells is shown. (b) The SH-SY5Y cells were pretreated with 1 mM LiCl for 1 hr in the absence or presence of 100 nM E2 and then challenged with 20 *μ*M 6-OHDA for 8 hr in control, 6-OHDA, 6-OHDA plus 1 mM LiCl, 6-OHDA plus 1 mM LiCl, and 100 nM E2 and 6-OHDA plus 100 nM E2 groups. (c) The SH-SY5Y cells were pretreated with 1 mM LiCl for 1 hr in the absence or presence of 100 nM E2 and then challenged with 20 *μ*M 6-OHDA for 8 hr in control, 6-OHDA, 6-OHDA plus 1 mM LiCl, 6-OHDA plus 1 mM LiCl, and 100 nM E2 and 6-OHDA plus 100 nM E2 groups. Western blotting of cleaved caspase-3 protein and quantification of each group are shown. *β*-Actin was used as an internal control for normalization. The value corresponds to the mean ± SEM of three independent experiments that have been done in triplicate (*n* = 9). ^*∗*^*p* < 0.05 compared with the control group; ^#^*p* < 0.05 compared with the 6-OHDA group.

We firstly examined the neuroprotective effect of the combination of lithium chloride and estradiol (E2) on the SH-SY5Y neuroblastoma cell line. The following tests evaluated the anti-apoptotic activity, including cell viability, TUNEL staining, cleavage caspase-3 mRNA, and protein expression. AlamarBlue was used to assess cell viability at 16 hr. The data showed that treatment of 20 *μ*Μ 6-OHDA significantly inhibited cell viability (100.0 ± 4.5% to 32.0 ± 5.3%). Moreover, 100 nM E2, 1 mM, or 5 mM LiCl treatment alone did not affect cell viability. The results demonstrated that 1 mΜ LiCl in the absence or presence of 100 nM E2 could significantly reverse 6-OHDA-induced neuronal toxicity (32.0 ± 5.3% to 60.7 ± 2.4%) (Figure 1(a)). Besides, the co-treatment of 1 mΜ LiCl plus 100 nM E2 showed better therapeutic efficacy than LiCl or E2 alone (32.0 ± 5.3% to 78.6 ± 3.3%). However, the higher dose of LiCl (5 mΜ) showed no neuroprotective activity either in the presence or absence of E2 (Figure 1(a)). In trypan blue assay, 20 *μ*Μ 6-OHDA treatment significantly inhibited cell viability (100.0 ± 9.4% to 25.5 ± 7.5%). The results demonstrated that 1 mΜ LiCl in the absence or presence of 100 nM E2 could significantly reverse 6-OHDA-induced neuronal toxicity (25.5 ± 7.5% to 63.5 ± 8.5%). The therapeutic effect of combination treatment was better than E2 or LiCl alone (Figure 1(b)). TUNEL was further used to detect DNA fragmentation during apoptosis. The data showed that treatment of 20 *μ*M 6-OHDA for 8 hr significantly increased TUNEL staining compared with the control group (3.5 ± 2.9% to 58.5 ± 6.4%), and the co-treatment of 1 mΜ LiCl plus E2 significantly reduced the number of TUNEL-positive cells (58.5 ± 6.4% to 10.5 ± 2.8%). Besides, the treatment of LiCl or E2 in the 6-OHDA-challenge group was 32.4% ± 6.5% and 22.9% ± 3.7%, respectively (Figure 2(a)). Then, we further checked the activated caspase-3 mRNA and protein expression in 6-OHDA-induced SH-SY5Y cells after the treatment of 6-OHDA for 8 hr. The data depicted that treatment of 1 mΜ LiCl or 100 nM E2 significantly attenuated 6-OHDA-induced upregulation of active caspase-3 expression (566.2 ± 33.5% to 390.4 ± 40.3%; 566.2 ± 33.5% to 453.4 ± 32.4%), respectively. Our result demonstrated that 1 mΜ LiCl plus 100 nM E2 significantly attenuated 6-OHDA-induced upregulation of cleaved caspase-3 mRNA expression (566.2 ± 33.5% to 222.5 ± 10.2%) (Figure 2(b)). The protein expression was also consistent with mRNA expression. The co-treatment of 1 mΜ LiCl plus 100 nM E2 significantly attenuated 6-OHDA-induced upregulation of cleaved caspase-3 protein expression (133.4 ± 5.4% to 70.3 ± 10.3%) (Figure 2(c)). In addition, we examined the upstream cascade included p-Akt, p-P38, and p-ERK expression.

### 3.2. Effect of LiCl plus Estradiol on 6-OHDA-Induced Downregulation of p-ERK and p-Akt And Upregulation of p-P38 Expression

**Figure 3:**
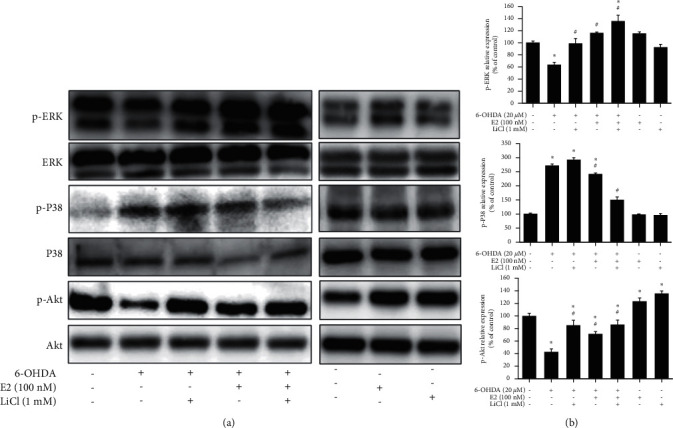
Effect of lithium chloride and E2 on 6-OHDA-induced downregulation of phospho-extracellular signal-regulated kinases (p-ERK), phospho-Akt (p-Akt), and p-P38 in SH-SY5Y cells. (a) SH-SY5Y cells were pretreated with 1 mM LiCl for 1 hr in the absence or presence of 100 nM E2 and then challenged with 20 *μ*M 6-OHDA for 1 hr. Western blotting for p-ERK, p-Akt, and p-P38 of the control, 6-OHDA, 6-OHDA plus 1 mM LiCl, 6-OHDA plus 100 nM E2, 6-OHDA plus 1 mM LiCl and 100 nM E2, 100 nM E2 alone and 1 mM LiCl alone groups. (b) The quantification results of p-ERK, p-Akt, and p-P38 relative density are shown. The value corresponds to the mean ± SEM of three independent experiments that have been done in triplicate (*n* = 9). ^*∗*^*p* < 0.05 compared with the control group; ^#^*p* < 0.05 compared with the 6-OHDA group.

The neuroprotective activity by co-treatment of LiCl plus E2 was proven to be mediated via the transient activation of the MAPK pathway. We confirmed the effects of LiCl plus E2 on p-Akt, p-ERK, and p-P38 expressions. The result depicted that 100 nM E2 or 1 mM LiCl treatment alone did not affect p-P38 and p-ERK expressions but increase in p-Akt expression (100.0 ± 4.6% to 123.4 ± 5.6%; 100.0 ± 4.6% to 135.7 ± 4.6%) respectively. Besides, the pretreatment 100 nM E2 or 1 mM LiCl in the 6-OHDA-challenge group could significantly reversed the 6-OHDA-induced down-regulation of p-Akt and p-ERK and enhanced 6-OHDA-induced upregulation of p-P38 expression. Further, the pretreatment of 100 nM E2 plus 1 mΜ LiCl significantly reversed 6-OHDA-induced downregulation p-ERK expressions and enhanced 6-OHDA-induced upregulation of p-P38 than each group (Figure 3(a)). The quantitative results also showed the same trend (Figure 3(b)). Except for apoptosis cascade, we also investigated about the neurogenesis effect of LiCl and E2 on neuronal cell.

### 3.3. Effect of LiCl plus Estradiol on Neurogenesis Factor Growth Associated Protein 43 (GAP43), N-myc, and TGase Protein Expression

**Figure 4:**
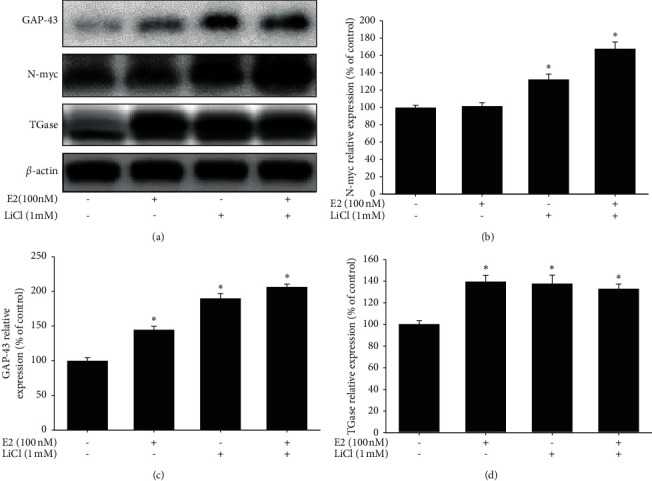
Effect of lithium chloride and estradiol on growth associated protein 43 (GAP-43), N-myc, and TGase in SH-SY5Y cells. (a) SH-SY5Y cells were co-treated with 1 mM LiCl in the absence or presence of 100 nM E2 for 24 hr. Western blotting for GAP-43 and N-myc of the control, 1 mM LiCl, 100 nM E2 and 1 mM LiCl plus 100 nM E2 groups. (b) The quantification result of GAP-43 relative density is shown. (c) The quantification result of TGase relative density is shown. (d) The quantification results of N-myc relative density are shown. *β*-Actin was used as an internal control for normalization. The value corresponds to the mean ± SEM of three independent experiments that have been done in triplicate (*n* = 9). ^*∗*^*p* < 0.05 compared with the control group; ^#^*p* < 0.05 compared with the 6-OHDA group.

We would like to examine further the neurogenesis-related marker that included growth-associated protein 43 (GAP43), N-myc, and TGase protein expression. Our data showed that the administration of 1 mM LiCl or 100 nM E2 could significantly increase the GAP-43, N-myc, and TGase expression (Figure 4(a)). Besides, the co-administration of LiCl and E2 significantly increase GAP-43 and N-myc protein expression than each group alone. The quantitative result also showed the same trend (Figures 4(b) and 4(c)). Thus, the result depicted that the co-treatment of LiCl and E2 may have a synergic effect on neurogenesis activity. Then, we would like to investigate further the neuroprotective activity *in vivo* zebrafish PD model.

### 3.4. Neuroprotective Effect of LiCl plus Estradiol on Zebrafish Locomotor Deficit

**Figure 5:**
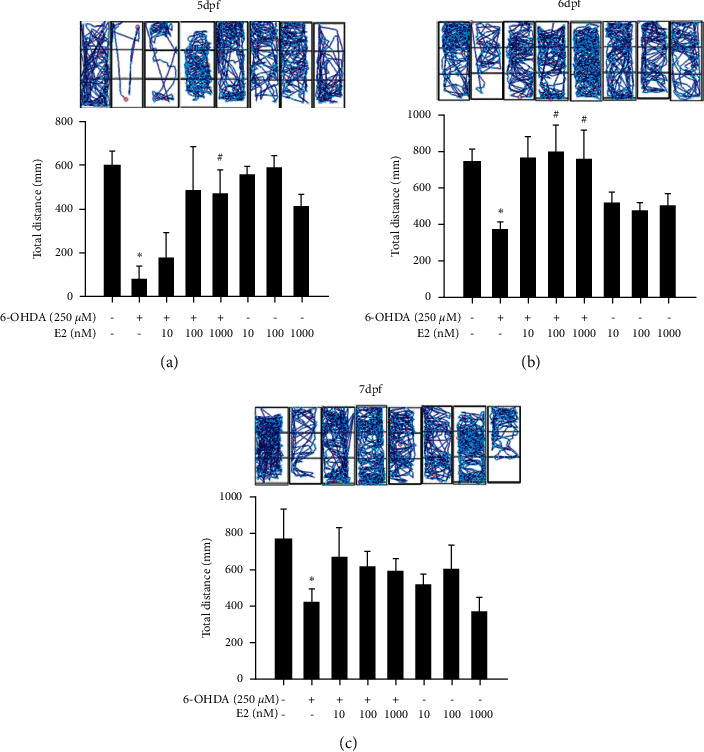
Effect of estradiol on 6-OHDA-induced locomotor deficit in the zebrafish PD model. Zebrafish were pretreated with 10, 100, and 1000 nM E2 from 9 hpf to 5 dpf and then challenged with 250 *μ*M 6-OHDA from 2 to 5 dpf. The locomotor behavior was measured at 5, 6, and 7 dpf of the control, 6-OHDA, 6-OHDA plus 10 nM E2, 6-OHDA plus 100 nM E2, 6-OHDA plus 1000 nM E2, 10 nM E2, 100 nM E2, and 1000 nM E2 groups. The upper panel demonstrates a representative swimming pattern, and the lower panel shows the average total swimming distance. The value corresponds to the mean ± SEM of two independent experiments that have been done in octuplicate (*n* = 16). ^*∗*^*p* < 0.05 compared with the control group; ^#^*p* < 0.05 compared with the 6-OHDA group.

**Figure 6:**
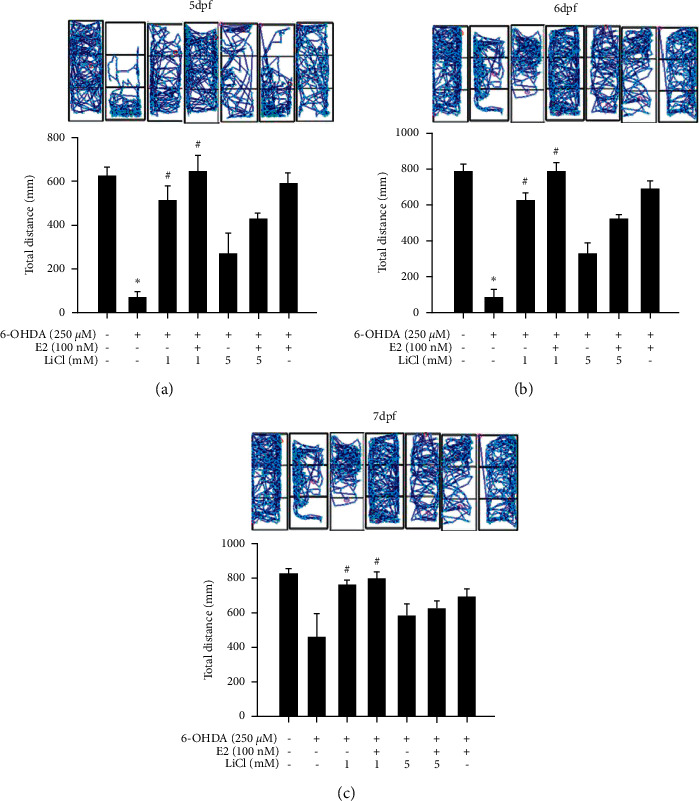
Effect of estradiol plus lithium chloride on 6-OHDA-induced locomotor deficit in the zebrafish PD model. Zebrafish were pretreated 1 or 5 mM LiCl in the absence or presence of 100 nM E2 from 9 hpf to 5 dpf and then challenged with 250 *μ*M 6-OHDA from 2 to 5 dpf of the the control, 6-OHDA, 6-OHDA plus 1 mM LiCl, 6-OHDA plus 100 nM E2, and 6-OHDA plus 1 mM LiCl and 100 nM E2 groups. The upper panel demonstrates a representative swimming pattern, and the lower panel shows the average total swimming distance. The value corresponds to the mean ± SEM of two independent experiments that have been done in octuplicate (*n* = 16). ^*∗*^*p* < 0.05 compared with the control group; ^#^*p* < 0.05 compared with the 6-OHDA group.

We then confirmed the locomotor activity and behavior of zebrafish in the zebrafish PD model. We firstly investigated the neuroprotective effect and dosage of E2 in zebrafish PD model. Zebrafish were pretreated with 10, 100, or 1000 nM E2 (9 hpf to 5 dpf) and in the absence or presence of 250 *μ*M 6-OHDA (2 to 5 dpf). We then analyzed zebrafish locomotor activity at 5, 6, and 7 dpf. The results showed that the treatment of 250 *μ*M 6-OHDA significantly decreased the total swimming distance of zebrafish in 5, 6, and 7 dpf (Figure 5). However, the pretreatment of 100 nM E2 significantly reversed 6-OHDA-induced down-regulation of total swimming distance in 6 and 7 dpf. The pretreatment of 1000 nM E2 only relived 6-OHDA-induced downregulation of total swimming distance at 5 dpf. Hence, we use 100 nM E2 in the follow-up experiment. We then examined the neuroprotective activity of the co-treatment of LiCl plus E2 in the same model. Zebrafish were pretreated with 1 or 5 mM LiCl plus 100 nM E2 (9 hpf to 5 dpf) and in the absence or presence of 250 *μ*M 6-OHDA (2 to 5 dpf). We also analyzed zebrafish locomotor activity at 5, 6, and 7 dpf. Our data demonstrated that administration of 1 mM LiCl with or without 100 nM E2 could significantly reverse 6-OHDA-induced downregulation of locomotor activity at 5, 6, and 7 dpf (Figure 6). Moreover, the combination treatment showed a synergic effect than alone groups only in 6 dpf but not in 5 and 7 dpf. However, the higher dosage of 5 mM LiCl with or without 100 nM E2 showed no neuroprotective effect in 5, 6, and 7dpf (Figure 6). Hence, we would like to use 1 mM LiCl for the further molecular marker examination.

### 3.5. Neurogenesis and Neuroprotective Effect of LiCl plus Estradiol on Zebrafish PD Model

**Figure 7:**
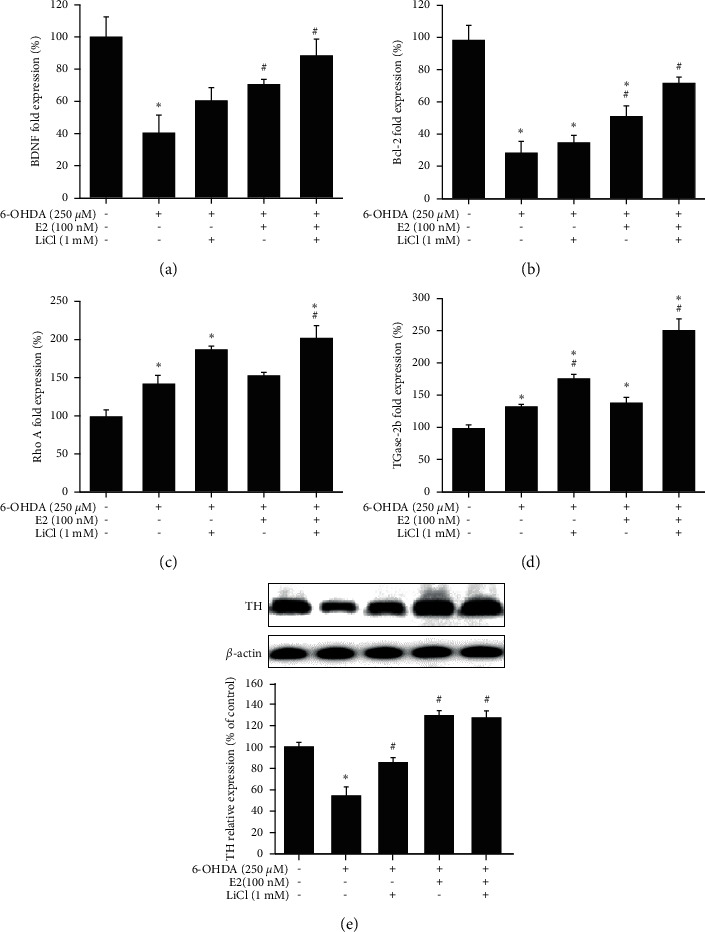
Neuroprotective effect of lithium chloride plus estradiol on the 6-OHDA-induced zebrafish PD model. (a) Zebrafish were pretreated with 1 mM LiCl (9 hpf to 5 dpf) in the absence or presence of 100 nM E2 and then challenged with 250 *μ*M 6-OHDA (2 to 5 dpf). Quantitative PCR of BDNF was performed. (b) Zebrafish were pretreated with 1 mM LiCl (9 hpf to 5 dpf) in the absence or presence of 100 nM E2 and then challenged with 250 *μ*M 6-OHDA (2 to 5 dpf). Quantitative PCR of Bcl-2 was performed. (c) Zebrafish were pretreated with 1 mM LiCl (9 hpf to 5 dpf) in the absence or presence of 100 nM E2 and then challenged with 250 *μ*M 6-OHDA (2 to 5 dpf). Quantitative PCR of Rho A was performed. (d) Zebrafish were pretreated with 1 mM LiCl (9 hpf to 5 dpf) in the absence or presence of 100 nM E2 and then challenged with 250 *μ*M 6-OHDA (2 to 5 dpf). Quantitative PCR of TGase 2b was performed. (e) Zebrafish were pretreated with 1 mM LiCl (9 hpf to 5 dpf) in the absence or presence of 100 nM E2 and then challenged with 250 *μ*M 6-OHDA (2 to 5 dpf). Western blotting of TH was performed. *β*-Actin was used as an internal control for normalization. The value corresponds to the mean ± SEM of three independent experiments that have been done in triplicate (*n* = 9). ^*∗*^*p* < 0.05 compared with the control group; ^#^*p* < 0.05 compared with the 6-OHDA group.

We confirmed the neurogenesis and neuroprotective effect of LiCl plus E2 in the zebrafish PD model with qPCR and western blotting. Zebrafish were pretreated with 1 mM LiCl plus 100 nM E2 (9 hpf to 5 dpf) and in the absence or presence of 250 *μ*M 6-OHDA (2 to 5 dpf). The qPCR and western blotting sample were collected at 5 dpf. We selected brain-derived neurotrophic factor (BDNF), Bcl-2 as apoptosis biomarker and transglutaminase 2b (TGase-2b), and Ras homolog family member A (Rho A) as neurogenesis biomarker. Our results demonstrated that treatment of 6-OHDA could significantly decrease BDNF and Bcl-2 mRNA expression (Figures 7(a) and 7(b)). At the same time, 6-OHDA could significantly increase TGase-2b and Rho A mRNA expression (Figures 7(c) and 7(d)). However, the treatment of E2 and LiCl plus E2 groups could significantly reverse 6-OHDA-induced downregulation of BDNF and Bcl-2. The combined group did not show a noticeable effect than alone groups because the effect of one compound is maximal. In addition, the LiCl plus E2 group enhanced 6-OHDA-induced upregulation of TGase-2b and Rho A mRNA expression and the other two groups (LiCl or E2 alone group) showed no significant effect in these two markers. Finally, we also use tyrosine hydroxylase (TH) as dopamine neuron biomarker. The data showed that 6-OHDA treatment could significantly decrease expression of TH from 100.0 ± 4.4% to 54.6 ± 8.4%. The pretreatment of LiCl, E2, and LiCl plus E2 could reverse 6-OHDA-induced downregulation of TH expression to 85.5 ± 4.6%, 130.7 ± 4.1%, and 127.9 ± 6.6%, respectively (Figure 7(e)).

**Figure 8:**
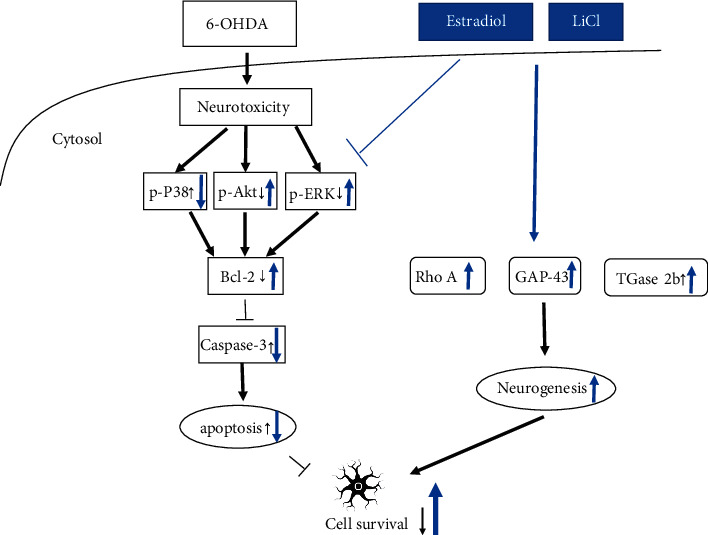
Schematic diagram of lithium chloride (LiCl) plus estradiol (E2) in 6-OHDA-induced neuronal death. 6-OHDA could induce cell apoptosis through increasing of p-Akt and p-ERK expression, decreasing p-P38, and further modulating the downstream apoptotic proteins, such as Bcl-2 and caspase-3. Our studies demonstrated that the co-treatment of LiCl and E2 could enhance neurogenesis via increasing Rho A, GAP-43, and TGase-2b protein expression. Besides, co-treatment of LiCl and E2 attenuated cell apoptosis through reversing 6-OHDA-induced downregulation of p-Akt, p-ERK and attenuating 6-OHDA-induced upregulation of p-P38 protein expression. The downstream cascade was also modulated, such as Bcl-2 and caspase-3. The 6-OHDA-induced upregulating Bcl-2 expression was reversed by the co-treatment of LiCl plus E2 and 6-OHDA-induced upregulating of caspase-3 expression was also attenuated. The neuroprotective effect of LiCl plus E2 significantly protected dopamine neurons against damage.

We summarize a putative diagram to describe the effect of LiCl plus E2 in menopausal PD (Figure 8). The treatment of 6-OHDA in neuron cell could increase p-P38, cleaved caspase-3. In zebrafish, treatment of 6-OHDA also decreases apoptotic related marker BDNF, Bcl-2, and increase neurogenesis related markers TGase-2b and Rho A mRNA expression. The treatment of 6-OHDA also decreases TH protein expression. However, the combination of LiCl and E2 alone could enhance neurogenesis proteins GAP-43 and N-myc in the neuronal cell. We also further confirmed the neurogenesis activity in zebrafish. LiCl and LiCl plus E2 could enhance 6-OHDA-induced upregulation of TGase-2b and Rho A mRNA expression. Besides, LiCl plus E2 also showed a synergic effect in anti-apoptotic activity. LiCl plus E2 protected SH-SY5Y cells and zebrafish against 6-OHDA-induced damage on neuron than LiCl or E2 alone groups via p-P38, p-Akt, Bcl-2, and caspase-3 cascade.

## 4. Discussion

Increasing reports depicted that estrogens may protect the nigrostriatal dopaminergic pathway affected in PD [43–45]. Animal studies and human clinical trials revealed that estrogen could increase dopamine synthesis and accelerate the metabolism of dopamine. Besides, the expression of dopamine receptors could also be affected by estrogen [46]. However, some clinical studies showed that the administration of HRT may exacerbate the PD symptoms and the side effects remain ambiguous [47]. Our studies intend to combine LiCl and E2 in the treatment of PD to enhance the neuroprotective activity of estrogen to improve the HRT. Except for our results, previous studies also combine lithium chloride with other reagents to enhance or increase its therapeutic efficacy. Some studies depicted that the combination of lithium chloride and agents could provide a synergic effect [48, 49]. Bai et al. also showed that LiCl and LY294002 (Akt inhibitor) co-treatment could increase osteogenesis activity and decrease osteoclast differentiation and bone absorption capacity compared with each group alone simultaneously. The co-treatment of LiCl and LY294002 significantly enhanced bone alkaline phosphatase levels and significantly decreased PINP, TRACP-5b, and CTX levels. The co-treatment also had the synergic effect [49]. Not only in animal science but also in plant science, Ali et al. demonstrated the synergic effect of LiCl and alkaline hydrogen peroxide in improving biomethanation at corn stalk [50]. The studies mentioned above all revealed that the feasibility of combination treatment of LiCl with other agents. Our study combined LiCl with estrogen and intends to enhance neuroprotective activity. LiCl plus E2 showed a synergic effect in neuroprotection than LiCl or E2 alone, respectively. The locomotor activity of zebrafish also revealed the same trend. The data also proved that the neuroprotective activity might come from neurogenesis and some reports also demonstrated the function of neurogenesis in PD.

Some research showed that increased neurogenesis could improve PD progress in *in vivo* or *in vitro* models [51–54]. Ferrer and Blanco showed that N-myc overexpression in the soma and nuclei of neurons vulnerable PD. The immunoreactivity of N-myc increased in subpopulations of reactive astrocytes in PD. These data suggest that N-myc and c-myc are involved in glial cell differentiation and growth and that this function may also apply to reactive astrocytes in human neurodegenerative disorders [52]. Although some literature reported N-myc as an oncogene in neuroblastoma [55, 56]. The recent study made by Shi in 2021 used N-myc as a biomarker of neurogenesis in SH-SY5Y cell model and other studies considered N-myc as an essential factor in neuronal differentiation. Besides, Hammerling et al. depicted that the mRNA expression of N-myc in SH-SY5Y was found not to change appreciably during the cell cycle and was also unaffected by proliferative inhibition induced by serum starvation or polyamine depletion. Treatment with TPA for 8 days could induce morphological and functional neuronal differentiation [57–59]. Except for N-myc, Park et al. found that the stimulation of IL-1*β* could lead to the differentiation of neuron cell and could upregulate Wnt5*α* protein expression via NF-*κ*B. The study also demonstrated that Rho A/ROCK/JNK pathway is involved in neurogenesis and use Rho A as a neurogenesis biomarker [60]. Lopez-Lopez et al. showed that fasudil could attenuate the L-DOPA-induced dyskinesia without decreasing the therapeutic effect of L-DOPA on motor behavior. Besides, the treatment of 6-OHDA significantly increases Rho A protein expression in rat striatum in PD model [61]. Our data showed the same trend that the treatment of 6-OHDA upregulates the expression of Rho A. We further confirmed that the pretreatment of LiCl plus E2 enhanced 6-OHDA-induced upregulation of Rho A expression. Li et al. also showed that the Pilose Antler Extracts (PAEs) reversed 6-OHDA-induced downregulation of the levels of monoamine neurotransmitters and amino acids in the striatum and cerebrospinal fluid in rat PD model. In addition, PAEs also increased the levels of GAP-43 in rat brain tissue, which represents that PAEs may facilitate neuronal growth and plasticity. The result also depicted that PAEs a possible mechanism for the protective effect of PAEs against 6-OHDA-induced neuronal cell death and may tightly correlate with the components in PAEs promoting neurogenesis [51]. Besides, O'Neil et al. also showed an AMPA receptor potentiator can provide neurochemical protection in the rodent PD model. This study also demonstrated that the administration AMPA receptor inhibitor was delayed until after cell death and accompanied by an increase in GAP-43 expression in the striatum provided tantalizing evidence for neurotrophic action [53]. Our data also showed that LiCl plus E2 could significantly increase GAP-43 expression, and the alone group could also do so. Except for the role of neurogenesis in PD progress, some studies also investigated the apoptosis cascade in PD progression.

Many studies demonstrated that the zebrafish animal model is a highly efficient high-throughput screening platform for neuroprotective activity [35, 62–65]. McGrath et al. listed zebrafish are increasingly used for early drug screening studies, especially approaches for assessing drug effects on apoptosis [62]. Cornin et al. showed that zebrafish could be a model suitable for high-throughput drug screening for PD. Their findings for minocycline and rasagiline echoed those studies in rodents and primates, and although, unlike studies in rodents, isradipine was not neuroprotective in zebrafish, which may better reflect the clinical situation. They showed that exposure of zebrafish larvae to 6-OHDA can be used to test for neuroprotection and neuro-restoration in a relatively high throughput manner [35]. Liao et al. also demonstrated that the ShK-like peptide PcShK3 has the ability to confer cardiovascular and neurological protective effects in a zebrafish model of drug screening. The peptide displayed an enjoyable neuroprotective activity that in combination with structurally guided dissection of peptides, PcShK3 suppressed the 6-OHDA-induced neurotoxicity on the locomotive behavior of zebrafish [63]. The same team also used the zebrafish PD model to examine the folded PpV*α* peptide. They showed the peptide protected PC12 cells against 6-OHDA-induced neurotoxicity via activating heme oxygenase-1 (HO-1) and attenuating inducible nitric oxide synthase (iNOS) protein expression. In vivo, PpV*α* peptide reversed the 6-OHDA-induced locomotor deficit of zebrafish and prevented the 6-OHDA-induced excessive ROS generation and subsequent dopaminergic neurons loss [64]. Kim et al. also revealed that pretreatment with MgT, and downstream upregulation of glutamate transporter EAAT4, has protective effects on neuronal survival, reduction in cerebral infarction, and preservation of learning and memory in zebrafish following hypoxia. This study also used zebrafish as an *in vivo* model to examine the neuroprotective activity which MgT could reversed 6-OHDA-induced locomotor deficit in zebrafish PD model [65]. Our study also used zebrafish PD model to examine the neuroprotective and neuro-regenerative activity of LiCl plus E2. The present study depicted that the combination of LiCl and E2 could show synergic protective effect against 6-OHDA damage and neuro-restorative effect than each treatment alone.

To our excitement, our present study shows several advantages of LiCl plus E2. We firstly applied the combination of LiCl plus E2 and intend to use the combination treatment to enhance the neuroprotective effect. The neuroprotective effect is possibly through anti-apoptosis and neurogenesis pathway via the synergic effect of LiCl plus E2. We hope that further experiments and observations could evaluate the specific mechanisms in order to contribute to the clinical application of potential treatments.

## 5. Conclusions

We firstly examined the neuroprotective activity of LiCl plus E2 in SH-SY5Y *in vitro* model. The data showed that the combination of LiCl and E2 revealed a better efficacy than LiCl or E2 alone. The results of the TUNEL stain and cleaved caspase-3 protein expression also showed the same trend. The 6-OHDA-induced increase of apoptotic cell number was significantly attenuated by the treatment of LiCl plus E2, and the effect of combination treatment is better than each group alone. LiCl plus E2 protected dopamine neurons against 6-OHDA damage via p-P38, p-Akt, and p-ERK cascade. Besides, LiCl plus E2 also enhanced SH-SY5Y cell neurogenesis related marker included GAP-43, N-myc protein expression. We further used *in vivo* zebrafish PD model to confirm the neuroprotective activity of LiCl plus E2. The data demonstrated that E2 could protect zebrafish against 6-OHDA damage in locomotor deficit. We further examined LiCl plus E2 in this model. To our excitement, the combination treatment significantly reversed 6-OHDA-induced downregulation of locomotor activity, which is better than each group alone. We also checked some neuroprotection (BDNF, Bcl-2, and TH) and neurogenesis factors (Rho A, TGase-2b) to verify the possible cascade of LiCl plus E2. Except for our results, previous studies also combine lithium chloride with other reagents to enhance or increase its therapeutic efficacy.

## Data Availability

The raw data supporting the conclusions of this manuscript will be made available by the authors, without undue reservation, to any qualified researcher.
